# Probabilistic Source Separation for Robust Fetal Electrocardiography

**DOI:** 10.1155/2013/109756

**Published:** 2013-12-01

**Authors:** Rik Vullings, Massimo Mischi

**Affiliations:** Department of Electrical Engineering, Eindhoven University of Technology, P.O. Box 513, 5600 MB Eindhoven, The Netherlands

## Abstract

Blind source separation (BSS) techniques are widely used to extract signals of interest from a mixture with other signals, such as extracting fetal electrocardiogram (ECG) signals from noninvasive recordings on the maternal abdomen. These BSS techniques, however, typically lack possibilities to incorporate any prior knowledge on the mixing of the source signals. Particularly for fetal ECG signals, knowledge on the mixing is available based on the origin and propagation properties of these signals. In this paper, a novel source separation method is developed that combines the strengths and accuracy of BSS techniques with the robustness of an underlying physiological model of the fetal ECG. The method is developed within a probabilistic framework and yields an iterative convergence of the separation matrix towards a maximum a posteriori estimation, where in each iteration the latest estimate of the separation matrix is corrected towards a tradeoff between the BSS technique and the physiological model. The method is evaluated by comparing its performance with that of FastICA on both simulated and real multichannel fetal ECG recordings, demonstrating that the developed method outperforms FastICA in extracting the fetal ECG source signals.

## 1. Introduction

Current fetal monitoring mainly relies on the cardiotocogram (CTG); the simultaneous registration of fetal heart rate; and uterine activity. Unfortunately, in many cases the information provided by the CTG is insufficient. In these cases, obstetricians have to rely on other sources of information or on their intuition and experience to make the optimal treatment plan. A valuable complementary source of information is provided by the fetal electrocardiogram (ECG) [1]. In clinical practice, the fetal ECG is measured during labor using an invasive electrode. The use of this electrode requires the fetal membranes to be ruptured and the cervix to be sufficiently dilated. An alternative method to obtain the fetal ECG makes use of electrodes placed on the abdomen of the mother [2].

Although these abdominal recordings are a promising candidate for use in fetal monitoring, their widespread use is impeded by the quality of the fetal ECG signals which is typically poor. Specifically, each signal recorded from the maternal abdomen consists of a mixture of signals, including the fetal ECG, maternal ECG, activity of abdominal muscles and uterus, and interferences from external sources. Several methods to extract the fetal ECG from such mixtures have been proposed in the literature [2–5]. Prominent among these methods are the so-called source separation methods such as independent component analysis (ICA) [2, 6]. Despite the successful application of these source separation methods for fetal ECG extraction, their use in clinical practice is limited to virtually none. The key reason for this is the relatively poor robustness in case of significant artifacts and noise [7]. In other words, in case the noninvasive fetal ECG recordings have relatively good quality, the source separation methods perform satisfactory and in case of poor signal quality, their performance breaks down.

One of the reasons for poor robustness with respect to signal quality lies in the fact that ICA assumes no *a priori* knowledge on the origin of the recorded signal mixtures. Hence, ICA-based methods are referred to as blind source separation (BSS) techniques. In case of noninvasive fetal ECG recordings, however, some knowledge on the source signals mixing is available. Exploitation of such knowledge can improve the robustness of the source separation, as demonstrated for audio signals by Knuth [8].

In this paper, we follow the approach by Knuth and develop a probabilistic framework to derive a generic source separation technique. This technique allows for inclusion of *a priori* knowledge on the mixing of the source signals. In case no a prior knowledge is included, this technique reduces to an ICA technique. To include prior knowledge in the source separation, we present a physiology-based probabilistic model that describes how fetal ECG signals mix to the noninvasive abdominal recordings. Our method, consequently, yields a probabilistic, physiology-based source separation technique for fetal ECG extraction.

In Section 2, our method is presented and the methodology for implementation and evaluation is discussed. In Section 3, the performance of our method is illustrated and evaluated by comparing it with the performance of a widely used ICA approach. In Section 4, we draw our conclusions.

## 2. Materials and Methods

### 2.1. Probabilistic Description of Source Separation Problem

When we assume a fetal ECG recording of *N* signals **x**(*t*), each comprising an unknown mixture of *M* source signals **s**(*t*), the goal of any source separation method is to unmix the recorded signals into the source signals:

(1) $$\begin{array}{c} x\left(t\right)=A\left(t\right)s\left(t\right), \end{array}$$

where **A**(*t*) is a [*N* × *M*] matrix, referred to as the mixing matrix. In the case of fetal ECG recordings, the time-dependent behavior of **A** originates from changes in the volume conductor between fetal heart and abdominal electrodes, for example, due to movement of either mother or fetus.

Using Bayes' rule, the probability that the source model of (1) is correct can be written as [9]

(2) $$\begin{array}{c} p\left(A,s\;|\;x\right)=\frac{p\left(x\;|\;A,s\right)p\left(\text{A},s\right)}{p\left(x\right)}, \end{array}$$

where we have omitted the time-dependency for reasons of clarity.

The expression in the denominator of (2) is referred to as the *evidence* and can be regarded as a normalization term, independent of the variables of interest (i.e., **A** or **s**). Hence, (2) can be simplified to

(3) $$\begin{array}{c} p\left(A,s\;|\;x\right)\propto p\left(x\;|\;A,s\right)p\left(A,s\right). \end{array}$$

The expression on the left-hand side of (3) is referred to as the *posterior*. The first term on the right-hand side of (3) is referred to as the *likelihood*. The second term is referred to as the *prior*.

As mentioned previously, the goal of the source separation method is to obtain the source signals **s**. Considering the source model in (1) with **x** known, inference of **A** also determines **s**. Since **A** is typically a smaller matrix than **s**, it is often computationally more efficient to estimate **A** rather than **s**. Hence, the problem of estimating the source signals **s** can be translated to the problem of estimating the mixing matrix **A**. In this context, the source signals **s** can be regarded as a nuisance parameter and omitted from the posterior by marginalization:

(4) $$\begin{array}{c} p\left(A\;|\;x\right)\propto p\left(A\right)\int p\left(x\;|\;A,s\right)p\left(s\right)\text{d}s. \end{array}$$

Here, **A** and **s** are assumed to be statistically independent, based on the assumption that the properties of signal propagation (i.e., the mixing matrix **A**) do not depend on the source signals and their magnitudes (i.e., the source matrix **s**).

In the context of this probabilistic description, the challenge of source separation methods is to infer **A** by maximizing the posterior probability distribution *p*(**A** | **x**) with respect to **A**. Inference on **A**, subsequently, provides an estimate for the source signals **s**(*t*).

### 2.2. Maximum A Posteriori Solution: Informed Source Separation

Until here, we have followed the descriptions of Knuth [8] and Bell and Sejnowski [10]. As Knuth shows in [8], prior knowledge—in terms of the probability distribution *p*(**A**)—can be included in the probabilistic model of (4) to yield a *Maximum A Posteriori* (MAP) solution of the source separation problem. Because of the inclusion of prior knowledge, this method is referred to as an informed source separation (ISS) method (as opposed to the BSS methods that do not allow for inclusion of prior knowledge). For fetal ECG estimation, this prior knowledge entails a mathematical description of the dipole nature of the ECG and of the propagation properties of the abdominal volume conductor [11].

#### 2.2.1. Mixing Model for Fetal ECG

When recorded relatively far away from the heart, the electrical activity of the heart can be approximated by an electrical dipole $\vec{\rho }(t)$ that changes in orientation and amplitude during the course of a cardiac cycle [12]. For a bipolar ECG recording between electrode *i* and a reference electrode *ℛ*, the electrical potential *V*_*i*_(*t*) recorded at the skin is accordingly given by

(5) $$\begin{array}{c} V_{i}\left(t\right)=\frac{1}{4\pi \epsilon }\left\{\frac{{\vec{r}}_{i}-{\vec{r}}_{f}}{{\left|{\vec{r}}_{i}-{\vec{r}}_{f}\right|}^{3}}-\frac{{\vec{r}}_{ℛ}-{\vec{r}}_{f}}{{\left|{\vec{r}}_{ℛ}-{\vec{r}}_{f}\right|}^{3}}\right\}\cdot \vec{\rho }\left(t\right), \end{array}$$

where *ϵ* describes the conductive properties of the medium between heart and electrode, which in this study are assumed to be the same for all recorded signals (i.e., the conductive properties are uniform), ${\vec{r}}_{i}$ describes the position of electrode *i*, ${\vec{r}}_{ℛ}$ describes the position of the reference electrode, and ${\vec{r}}_{f}$ describes the position of the fetal heart.

The electrical dipole $\vec{\rho }(t)$ is also known as the vectorcardiogram (VCG) and represents 3 orthogonal ECG sources that, when combined together, can describe any recorded ECG signal. In the context of source separation, $\vec{\rho }$ acts as the source matrix **s** in (1). Since the electrical potentials from the skin surface **V** correspond to **x**, the mixing matrix for fetal ECG can be described as

(6) $$\begin{array}{c} A_{ij}=\frac{1}{c}\left\{\frac{r_{ij}-r_{fj}}{{\left|{\vec{r}}_{\text{i}}-{\vec{r}}_{f}\right|}^{3}}-\frac{r_{ℛj}-r_{fj}}{{\left|{\vec{r}}_{ℛ}-{\vec{r}}_{f}\right|}^{3}}\right\}, \end{array}$$

where *c* = 4*πϵ*.

We can rewrite (6) to

(7) $$\begin{array}{c} A_{ij}=\frac{r_{ij}-r_{ℛj}}{c{\left|{\vec{r}}_{i}-{\vec{r}}_{f}\right|}^{3}}-\frac{r_{ℛj}-r_{fj}}{c}\left\{\frac{{\left|{\vec{r}}_{i}-{\vec{r}}_{f}\right|}^{3}-{\left|{\vec{r}}_{ℛ}-{\vec{r}}_{f}\right|}^{3}}{{\left|{\vec{r}}_{i}-{\vec{r}}_{f}\right|}^{3}{\left|{\vec{r}}_{ℛ}-{\vec{r}}_{f}\right|}^{3}}\right\}. \end{array}$$

In this expression, the first term on the right-hand side involves a (scaled) version of the difference in position between the abdominal electrodes *i* and *ℛ*. When we would assume that the distance between the fetal heart and the various electrodes is approximately the same, or at least that the difference between these heart-electrode distances is much smaller than the heart-electrode distance itself, the term between braces on the right-hand side of (7) tends to zero and, accordingly, we can simplify (7) to

(8) $$\begin{array}{c} A_{ij}=\frac{r_{ij}-r_{ℛj}}{c{\left|{\vec{r}}_{i}-{\vec{r}}_{f}\right|}^{3}}+\eta_{ij}. \end{array}$$

Here, *η*_*ij*_ is a noise term that expresses our model inaccuracies, such as the assumption to ignore the second term on the right-hand side of (7). Based on the central limit theorem, *η*_*ij*_ is taken to be a Gaussian noise term, with variance *σ*_*ij*_^2^ and mean *μ*_*ij*_. Based on our geometric assumptions leading to (8), we assume *μ*_*ij*_ to be zero.

Finally, to facilitate an analytical solution to the source separation problem, we ignore the dependence of the scaling $\alpha_{i}=1/c{\left|{\vec{r}}_{i}-{\vec{r}}_{f}\right|}^{3}$ on ${\vec{r}}_{i}$:

(9) $$\begin{array}{c} A_{ij}=\alpha_{i}\left(r_{ij}-r_{ℛj}\right)+\eta_{ij}. \end{array}$$

#### 2.2.2. Prior Probability Distribution

We can express our belief in the mixing model of (9) in terms of the prior probability distribution:

(10) $$\begin{array}{c} p\left(A\;|\;\sigma \right)=\int p\left(A,\vec{\alpha }\;|\;\sigma \right)p\left(\vec{\alpha }\;|\;\sigma \right)\text{d}\vec{\alpha }. \end{array}$$

Here, we have assumed the variance *σ*_*ij*_^2^ to be known. We will address the estimation of *σ*_*ij*_^2^ in Section 2.3.2.

When, for reasons of mathematical simplification, we assume the elements of the mixing matrix to be mutually independent, we can write $p\left(A,\vec{\alpha }|\sigma \right)$ as

(11) $$\begin{array}{c} p\left(A,\vec{\alpha }\;|\;\sigma \right)\approx \underset{ij}{\prod }p\left(A_{ij},\alpha_{i}\;|\;\sigma_{ij}\right), \end{array}$$

with, compare (9),

(12) p(Aij,αi ∣ σij)  =12πσij2exp⁡{−12σij2[Aij−αi(rij−rℛj)]2}.

This assumption of mutual independence of the elements of **A** is weak and can potentially reduce the performance of the source separation. More specifically, information on the mixing of ECG signals for one electrode provides information on the mixing for another electrode, since the electrode positions are known.

For the other conditional probability distribution in (10), we know that $\vec{\alpha }$ represents the scaling of ECG signals per electrode. This scaling depends on tissue propagation properties and the distance between fetal heart and electrode. The scaling must be positive and an upper and lower limit *a*_1_ and *a*_2_, respectively, can be defined based on the possible distances between heart and electrodes as well as on studies on tissue propagation properties [13]. Taking the probability distribution for $\vec{\alpha }$ to be uniformly distributed between these limits and, analogously to considerations above, assuming the elements of $\vec{\alpha }$ to be mutually independent, *p*(*α*_*i*_ | *σ*_*ij*_) can be written as

(13) $$\begin{array}{c} p\left(\alpha_{i}\;|\;\sigma_{ij}\right)=\{\begin{array}{cc} \frac{1}{a_{2}-a_{1}} & \text{for}\;\;a_{1}\leq \alpha_{i}\leq a_{2}\\ 0 & \text{otherwise}. \end{array} \end{array}$$

Combining (10)–(13) and solving the integral in (10) give

(14) p(A ∣ σ)=∏ij∫a1a212πσij2(a2−a1)  ×exp⁡{−12σij2[Aij−αi(rij−rℛj)]2}dαi=∏ijerf⁡[fij(a1)]−erf⁡[fij(a2)]2(a2−a1)(rij−rℛj),

where

(15) $$\begin{array}{c} \mathrm{erf}\left(z\right)=\frac{2}{\sqrt{\pi }}\overset{z}{\underset{0}{\int }}\exp\left(-t^{2}\right)\text{d}t,\\ f_{ij}\left(a_{k}\right)=\frac{A_{ij}-a_{k}\left(r_{ij}-r_{ℛj}\right)}{\sqrt{2}\sigma_{ij}}. \end{array}$$

#### 2.2.3. Dealing with Multiple Sources

As mentioned in Section 1, noninvasive fetal ECG recordings consist of a mixture of source signals of which the three orthogonal fetal ECG components **S** comprise only three source signals. We can estimate the other sources using a standard implementation of ICA. Specifically, we can assume no prior knowledge for these sources and include this lack of knowledge in our probability distribution for **A**:

(16) $$\begin{array}{c} p\left(A_{ij}\;|\;\sigma_{ij}\right)=\{\begin{array}{cc} \frac{\mathrm{erf}\left[f_{ij}\left(a_{1}\right)\right]-\mathrm{erf}\left[f_{ij}\left(a_{2}\right)\right]}{2\left(a_{2}-a_{1}\right)\left(r_{ij}-r_{ℛj}\right)} & \text{for}\;\;i\leq 3\\ \text{constant} & \text{else}. \end{array} \end{array}$$

#### 2.2.4. Towards a Solution

When we consider the posterior probability distribution of (4), we should realize that inference on the mixing matrix **A** entails maximization of the posterior probability with respect to **A**. Moreover, due to the fact that the logarithm is a monotonically increasing function, maximization of *p*(**A** | **x**) yields the same optimal value for **A** as would maximization of log⁡*p*(**A** | **x**).

As a first step to solve the inference problem, we follow Bell and Sejnowski [10] and assume the source signals **s** to be mutually independent. Furthermore, we express our belief in (1) by assigning a delta function to the likelihood *p*(**x** | **A**, **s**). Combining these assumptions with the posterior probability in (4) gives

(17) $$\begin{array}{c} p\left(A\;|\;x\right)\propto p\left(A\right)\int \overset{N}{\underset{i}{\prod }}\delta \left(x_{i}-\overset{M}{\underset{k}{\sum }}A_{ik}s_{k}\right)\overset{M}{\underset{j}{\prod }}p_{j}\left(s_{j}\right)\text{d}s, \end{array}$$

which can be solved to

(18) $$\begin{array}{c} p\left(A\;|\;x\right)\propto \frac{p\left(A\right)}{\det A}\underset{m}{\prod }p_{m}\left(\underset{k}{\sum }W_{mk}x_{k}\right), \end{array}$$

with **W** being the inverse of **A**, referred to as the unmixing matrix.

Taking logarithms on either side gives

(19) log⁡p(A ∣ x)=log⁡p(A)+logdetW+∑mlog⁡pm(∑kWmkxk)+C.

Here, *C* is a constant, independent of **A**.

For clarity, we introduce the estimated sources $\hat{s}$ as $\hat{s}=Wx$. Furthermore, instead of maximizing the posterior probability distribution with respect to **A**, we maximize it with respect to the separation matrix **W** [8]. Accordingly,

(20) ∂∂Wijlog⁡p(A ∣ x)=∂∂Wijlog⁡p(A)+Aji+xj((∂/∂s^i)pi(s^i)pi(s^i)),

or in terms of the derivative with respect to the matrix **W**:

(21) $$\begin{array}{c} \frac{\partial }{\partial W}\log p\left(A\;|\;x\right)=-A^{T}MA^{T}+A^{T}+\left(\frac{\left(\partial /\partial {\hat{s}}_{i}\right)p_{i}\left({\hat{s}}_{i}\right)}{p_{i}\left({\hat{s}}_{i}\right)}\right)x^{T}, \end{array}$$

where

(22) Mij=∂∂Wijlog⁡p(A)={2πσij2exp⁡[fij2(a1)]−exp⁡[fij2(a2)]erf⁡[fij(a1)]−erf⁡[fij(a2)]for  i≤30else.

The optimal unmixing matrix **W** can be inferred by implementing a gradient search algorithm. Moreover, by postmultiplying (21) by **W** ^*T*^ **W**, the expression can be made invariant to changes in the definition of the coordinate system that defines the various $\vec{r}$ [14]:

(23) $$\begin{array}{c} \Delta W=-A^{T}MW+W+\left(\frac{\left(\partial /\partial {\hat{s}}_{i}\right)p_{i}\left({\hat{s}}_{i}\right)}{p_{i}\left({\hat{s}}_{i}\right)}\right){\hat{s}}^{T}W, \end{array}$$

where Δ**W** is the update term for the gradient search algorithm:

(24) $$\begin{array}{c} W_{i+1}=W_{i}+\lambda \Delta W, \end{array}$$

with *λ* as the learning rate.

### 2.3. Implementation Challenges

#### 2.3.1. Numerical Accuracy of Error Function

When implementing the proposed source separation method, singularities can arise due to a finite numerical accuracy in estimating the error functions in the denominator of (22). Specifically, we implemented the source separation in MATLAB (The MathWorks Inc.), which can no longer distinguish between error functions when the arguments have exceeded the value of 6 (or are smaller than −6):

(25) $$\begin{array}{c} \mathrm{erf}\left(z_{1}\right)-\mathrm{erf}\left(z_{2}\right)=0,\;\text{if}\;\;z_{1}\geq 6\;\wedge \;z_{2}\geq 6. \end{array}$$

To avoid such singularities, we can approximate the error function by [15]

(26) $$\begin{array}{c} \mathrm{erf}\left(z\right)=\frac{z}{\left|z\right|}\left[1-\frac{1}{{\left(\sum_{i=0}^{4}b_{i}{\left|z\right|}^{i}\right)}^{4}}\right], \end{array}$$

with *b*_0_ = 1, *b*_1_ ≈ 0.28, *b*_2_ ≈ 0.23, *b*_3_ ≈ 9.8 · 10^−4^, and *b*_4_ ≈ 7.8 · 10^−2^.

Implementation of this approximation in MATLAB resolves the issue with finite numerical accuracy of the error function, no longer yielding zero difference when both *z*_1_ and *z*_2_ exceed 6 but are not identical.

#### 2.3.2. Estimating Model Inaccuracies

In Section 2.2.2, we have assumed the variance **σ** to be known. When analyzing (23), the term **A** ^*T*^ **M** **W** acts as a physiological component. The other terms together entail a typical BSS solution. In other words, **A** ^*T*^ **M** **W** is a physiology-based correction to the source separation. According to (22), the degree of correction is controlled by **σ**. Large values for **σ** indicate little confidence in the prior, physiology-based model and cause the method to act more or less as a BSS approach. Large confidence in the prior model, represented by small values for **σ**, results in a larger contribution of the prior model to the source separation.

For fetal ECG recordings, inaccuracies in the prior model arise from noise in the ECG signals or from erroneous assumptions with respect to the uniform propagation properties of the volume conductor or with respect to the sphere-like shape of the pregnant abdomen with the fetal heart in the center. These model inaccuracies can be tested by using the prior model to estimate $\vec{\rho }(t)$ and, subsequently, inverting the model to estimate the ECG signals from the estimated $\vec{\rho }(t)$:

(27) $$\begin{array}{c} \vec{\rho }\left(t\right)=A^{†}V\left(t\right),\\ \hat{V}\left(t\right)=A\vec{\rho }\left(t\right). \end{array}$$

Here, **A** ^†^ indicates the pseudoinverse of **A**, and **A** is provided by the prior model of (9).

The difference signal *ϵ*(*t*) between the recorded and estimated ECG signals yields an indication for the model accuracy. Specifically, the variance of *ϵ* can be used as a measure for **σ** [16]:

(28) $$\begin{array}{c} \sigma_{ij}^{2}=\text{var}\left[V_{i}\left(t\right)-{\hat{V}}_{i}\left(t\right)\right]. \end{array}$$

This expression implicitly assumes that *σ*_*ij*_ is independent of *j* (i.e., *σ*_*ij*_ = *σ*_*i*_).

To account for changes in the circumstances during the fetal ECG recording, for example, when the mother is having uterine contractions, the variance is determined within a sliding window of 2 seconds.

### 2.4. Evaluation of Source Separation

The developed probabilistic source separation method is evaluated by assessing its performance in extracting fetal ECG source signals from noninvasive recordings. The performance is evaluated by comparing it with that of a widely used ICA method: FastICA [17]. The performance of the source separation techniques is assessed both quantitatively and qualitatively. Quantitative assessment is achieved using simulated recordings and qualitative assessment is done via actual abdominal recordings.

#### 2.4.1. Data

**Figure 1:**
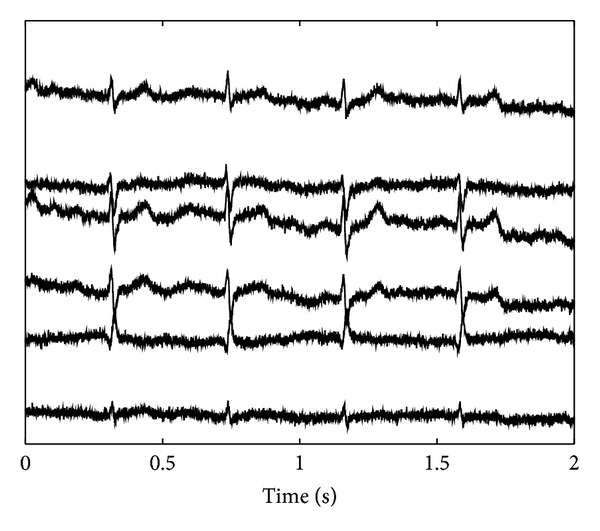
Example of a simulated 6-channel fetal ECG recording.

*Simulated Fetal ECG Recordings.* For simulating fetal ECG recordings, we use three orthogonal ECG signals recorded from an adult. These ECG signals are linearly combined, via a randomly determined mixing matrix, into *N* ECG signals. To mimic the low quality of fetal ECG signals, these *N* ECG signals are corrupted by muscular interferences and noise, obtained via a *N*-channel recording of bipolar measurements on a subject's arm. These measurements contain muscle activity and noise but due to their bipolar nature and position contain virtually no ECG contribution. Finally, also artificial powerline interference is added. An example of a simulated fetal ECG recording is shown in Figure 1.

To evaluate the developed source separation method for various degrees of signal quality, the signal to noise ratio (SNR) is varied between −10 and +30 dB. For each SNR, the evaluation is repeated 20 times to suppress the influence of the randomly determined mixing matrix. That is, in each repetition the mixing matrix is determined by picking its coefficients from a Gaussian distribution with unit variance.

**Figure 2:**
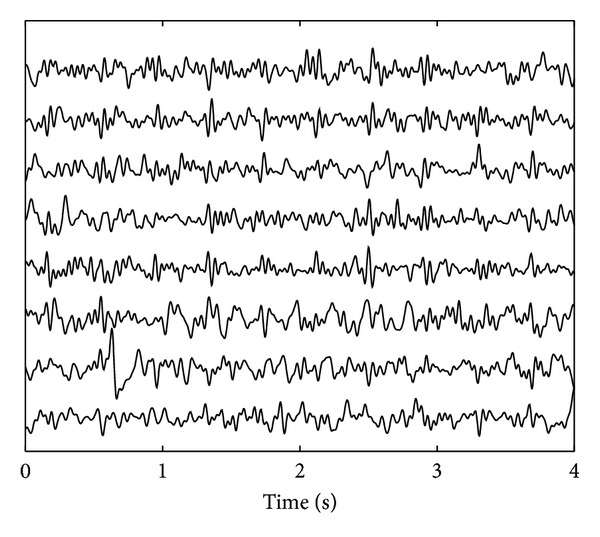
Example of real 8-channel fetal ECG recording. In this recording, the maternal ECG has already been removed using an adaptive template subtraction method [5] and high-frequency components of muscular activity have been suppressed by bandpass filtering between 1 and 70 Hz.

*Real Noninvasive Fetal ECG Recordings.* The noninvasive fetal ECG recordings are performed at the Máxima Medical Center, Veldhoven, The Netherlands, using a NEMO data acquisition system (NEMO Healthcare BV, The Netherlands), after having obtained written informed consent from the mother. At the time of recording, the mother was 28 weeks pregnant. Eight abdominal signals are acquired simultaneously with a 1 kHz sampling rate and are preprocessed to suppress powerline interference, muscle activity, and maternal ECG according to [5]. An example of the abdominal recording after preprocessing is depicted in Figure 2. It should be noted here that the preprocessing, by far, does not yield a good-quality fetal ECG and, hence, the need for additional processing, for example, by source separation. It should be noted as well that many others have applied (blind) source separation techniques without prior removal of the maternal ECG [2, 6]. In that case, the source separation technique, besides fetal ECG, also attempts to estimate the maternal ECG. As a result, fewer sources remain to estimate other interferences as well (i.e., the number of sources that can be extracted is restricted to the number of recorded signals: *M* ≤ *N*) yielding the fetal ECG sources to be more likely a mixture of fetal ECG and interferences.

#### 2.4.2. Evaluation Criteria

In our simulations, the performance of the source separation methods is quantified in terms of the normalized mean squared error *e* between the original sources **S** and the estimated sources $\tilde{S}$:

(29) $$\begin{array}{c} e=\frac{\sum \left(\tilde{S}-S\right){\left(\tilde{S}-S\right)}^{T}}{\sum SS^{T}}. \end{array}$$

The performance in separating sources in actual fetal ECG recordings is determined by assessing the ability of a peak detection algorithm to determine the fetal heart rate. The employed peak detection algorithm is based on a continuous wavelet transform [18] and the source signal representing the fetal ECG is selected based on visual inspection. The ability to correctly detect fetal ECG peaks is quantified by the sensitivity (Se):

(30) $$\begin{array}{c} \text{Se}=\frac{\text{TP}}{\text{TP}+\text{FN}}, \end{array}$$

and positive predictive value (PPV):

(31) $$\begin{array}{c} \text{PPV}=\frac{\text{TP}}{\text{TP}+\text{FP}}. \end{array}$$

Here, TP (true positive) is the number of correctly detected peaks, FP (false positive) the number of detections that were incorrectly labeled as ECG peaks, and FN (false negative) the number of ECG peaks that were missed by the detection algorithm. As a gold standard for assessing whether peaks are detected correctly, visual annotation by a clinical expert, on fetal ECG recordings in which the maternal ECG has been suppressed, is used.

With fetal heart rate detected, further enhancement of the fetal ECG can be achieved by (adaptively) averaging various consecutive ECG complexes, for example, as described in [16].

## 3. Results and Discussion

### 3.1. Results on Simulated Data

**Figure 3:**
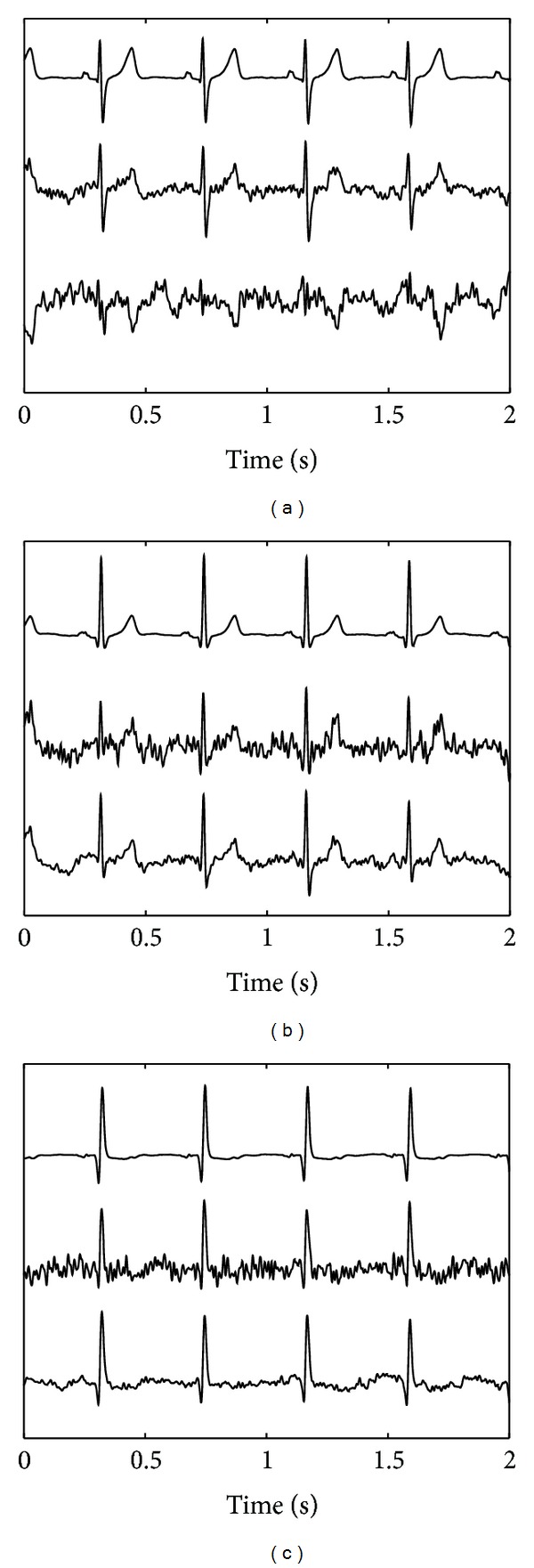
Results of source separation by the developed ISS method and FastICA for SNR of 6 dB. Each panel represents one of the three orthogonal ECG sources. In each panel, the top line represents the ECG source used in the simulation, the center line represents the corresponding source extracted by the ISS method, and the bottom line represents the corresponding source extracted by FastICA. The simulated fetal ECG recording used in the source extraction was depicted in Figure 1.

In Figure 3, an example of the source extraction by both the developed ISS method and FastICA is shown. The sources are extracted from the simulated fetal ECG recording that was depicted in Figure 1 and that has a SNR of 6 dB. As mentioned earlier, three orthogonal ECG sources were used to simulate the *N* = 6 channels of the fetal ECG recording. For each of the orthogonal ECG sources, the extracted source that matches the ECG best, in terms of least squared error, is used for the evaluation.

**Figure 4:**
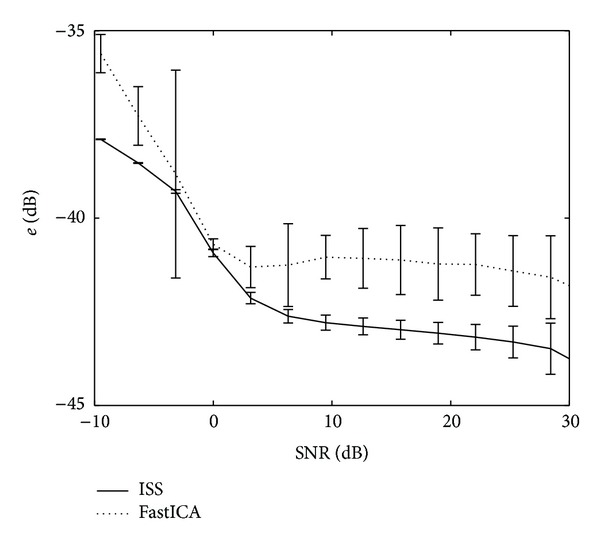
Performance of both source separation methods as a function of the SNR of the simulated recordings. Each depicted data point is the mean over 20 simulations with random mixing matrix. The standard deviations over these 20 simulations, although often too small to see in the graph, are also plotted.

In Figure 4, the performance of both source separation methods as a function of the SNR of the simulated fetal ECG recordings is depicted. This figure shows that the developed ISS method outperforms FastICA for all SNR.

### 3.2. Results on Real Data

**Figure 5:**
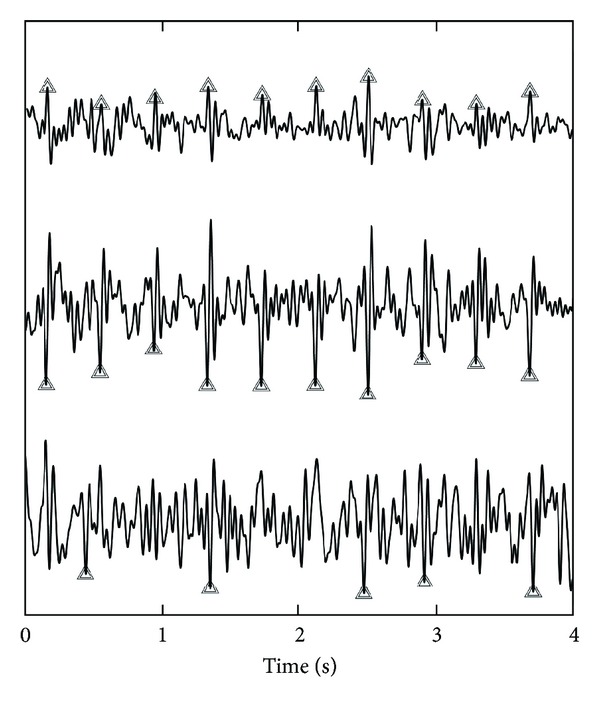
The upper plot shows a recorded and annotated fetal ECG signal. This depicted signal was preprocessed as described in Section 2.4.1 and was visually selected out of the *N* = 8 recorded signals as the one with the best fetal ECG. The center plot shows the fetal ECG source signals estimated with the developed ISS method. The lower plot shows the fetal ECG source signal estimated with FastICA. The triangles indicate detected fetal QRS complexes.

In Figure 5, the performance of the source separation methods for real fetal ECG recordings is depicted. The upper plot shows one of the recorded fetal ECG signals and the center plot shows the best fetal ECG source determined by ISS. The lower plot shows the best fetal ECG source by FastICA. The best ECG source is hereby defined as the source that yields the highest Se and PPV for ECG peak detection. It has to be noted here that the annotation of the recorded fetal ECG signal was based on visual analysis of all recorded channels at the same time. ECG peaks that are not distinctively discernible in the depicted ECG signal might have been annotated based on a different channel.

In total, 1532 ECG peaks have been annotated by the clinical expert in the 10-minute long abdominal recording. The performance of the employed peak detection algorithm in finding all these peaks in the ISS estimated fetal ECG source signal is Se = 0.88 and PPV = 0.84. In the FastICA estimated source signal, the performance of the peak detection is Se = 0.72 and PPV = 0.78.

### 3.3. Discussion

As mentioned in Section 2.3.2, when considering the update rule for the developed ISS method in (23), it shows that the last two terms on the right-hand side correspond to the ICA method developed by Bell and Sejnowski [10]. The first term on the right-hand side is a correction to the ICA solution and is based on *a priori* knowledge of the electrophysiology of the ECG. Any solution to the source separation problem by ISS, hence, entails a tradeoff between the ICA solution and the solution based on the physiological model of Section 2.2.1. The ratio in which each of these solutions contributes to the ISS solution strongly depends on estimated model inaccuracies: **σ**, compare (22).

The abovementioned tradeoff between ICA and physiology can also be regarded as a tradeoff between accuracy and robustness. We have illustrated this by applying our method to high-quality, yet realistic, (simulated) fetal ECG recordings and to lower-quality, but commonly more frequently encountered, (real) fetal ECG recordings. The statement of lower quality for the real recordings is based on visual comparison of Figures 1 and 2. The simulated recordings show that the gained robustness (ISS can locate all three fetal ECG sources, while FastICA could only estimate the two sources in Figures 3(b) and 3(c)) comes at the expense of a loss in accuracy; the three sources by ISS contain more noise than the two sources by FastICA. With regard to accuracy, as shown in Figure 4, the lack of accuracy—in terms of more noise in the estimated sources—by the ISS method is limited and is outweighed by the increase in robustness, as evidenced by the better performance of ISS for all SNR. With regard to robustness, as shown in Figure 5, for lower-quality fetal ECG recordings, the lack of robustness in BSS methods causes FastICA to fail in estimating a proper fetal ECG source, while the more robust ISS method can still estimate a fetal ECG source. In terms of detecting the fetal heart rate in the estimated sources, ISS outperforms FastICA by a sensitivity increase of 0.16 and an increase in the positive predictive value of 0.06. As already indicated, when comparing the sources extracted from the simulated data in Figure 3, it shows that—although ISS can better reproduce the original sources in terms of ECG shape—the ISS sources contain more noise than the FastICA sources. The main reason for this is that FastICA is not capable of estimating, in particular, the first ECG source (Figure 3(a)). As a result, for the 6-channel recording, FastICA has 4 sources remaining that represent noise. The ISS method estimates all three ECG sources and hence has only 3 sources remaining to represent the noise. By increasing the number of recorded signals *N* and therefore the number of possible sources *M* that can be estimated, the accuracy of the source separation can be improved. In addition, when the goal of the source separation method is to compute fetal heart rates, the morphology of the extracted sources is more important than the noise in these sources. For example, Figure 3(a) shows that the ISS method can better preserve this morphology than FastICA.

In this paper, the developed ISS method was applied to extract fetal ECG sources from preprocessed abdominal recordings. This preprocessing includes suppression of the maternal ECG and in this paper we used a template-based method to do this. As an alternative approach, others have used BSS techniques to extract fetal ECG sources directly from (unprocessed) abdominal recordings [2, 19]. The developed ISS method can also be applied directly on the unprocessed recordings, but for this approach to perform up to its potential a proper prior model of the maternal ECG has to be included next to the model for the fetal ECG. This extension of our ISS method is subject for further studies.

## 4. Conclusions

In this paper, a source separation technique for fetal ECG signals was developed that exploits prior knowledge on the signal mixing. When critically examining the presented solution to the source separation problem, it shows that the developed technique is similar to the Bell and Sejnowski [10] ICA approach, but with a correction that pushes the separation matrix towards a physiological model of the fetal ECG. The confidence in this physiological model determines the degree of correction. Little confidence causes the method to act as BSS technique with little to no pushing towards the physiological model. More confidence, on the other hand, leads to a technique that is more governed by the physiological model.

With respect to FastICA, the developed method performs better in retrieving the ECG sources in simulated and real fetal ECG recordings. More extensive evaluation of the developed method is however required to conclusively state about its performance, for example, in case of poorly determined electrode positions.
